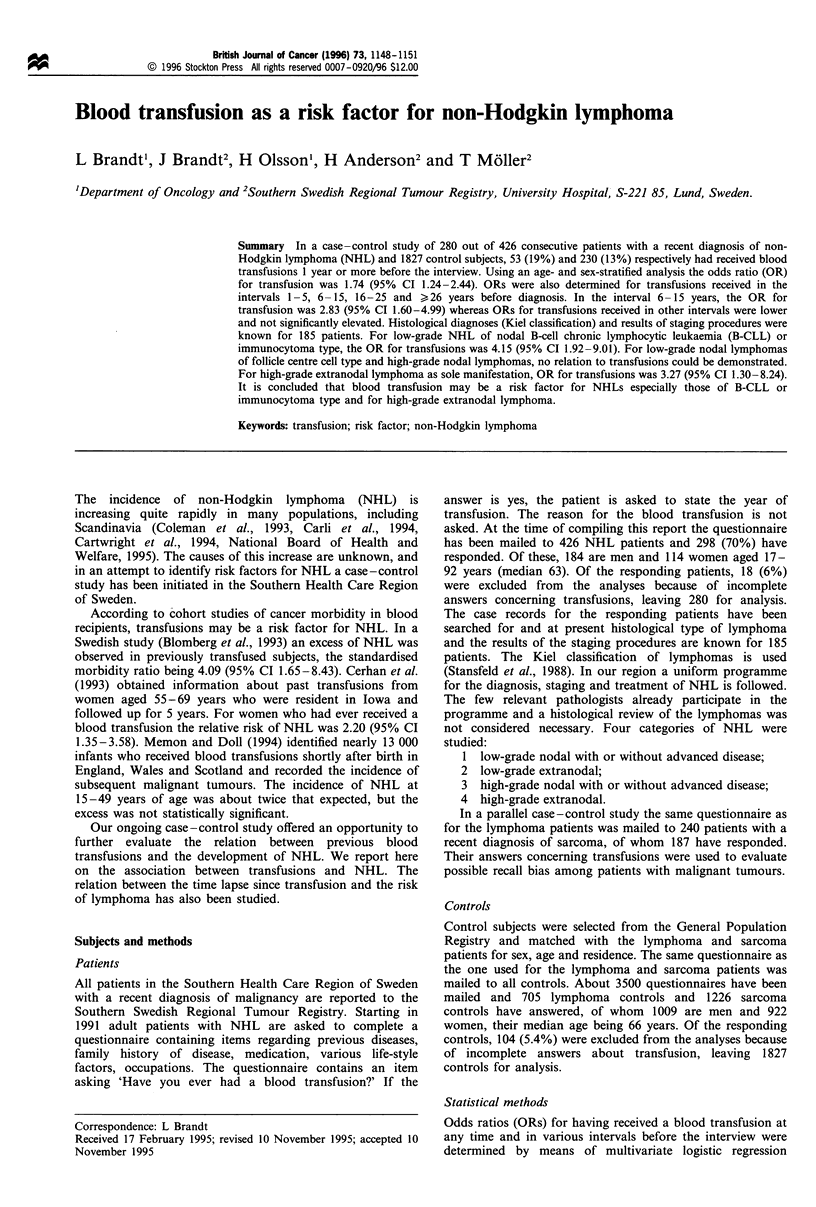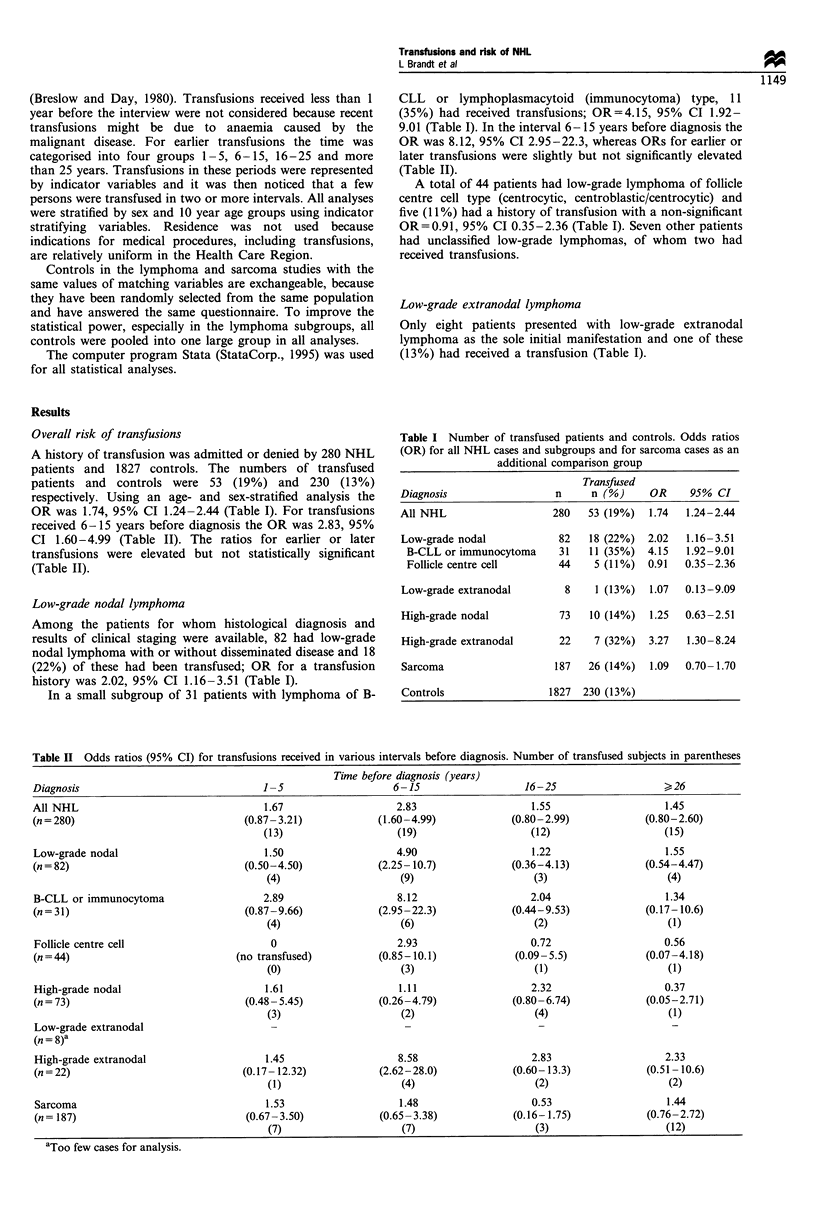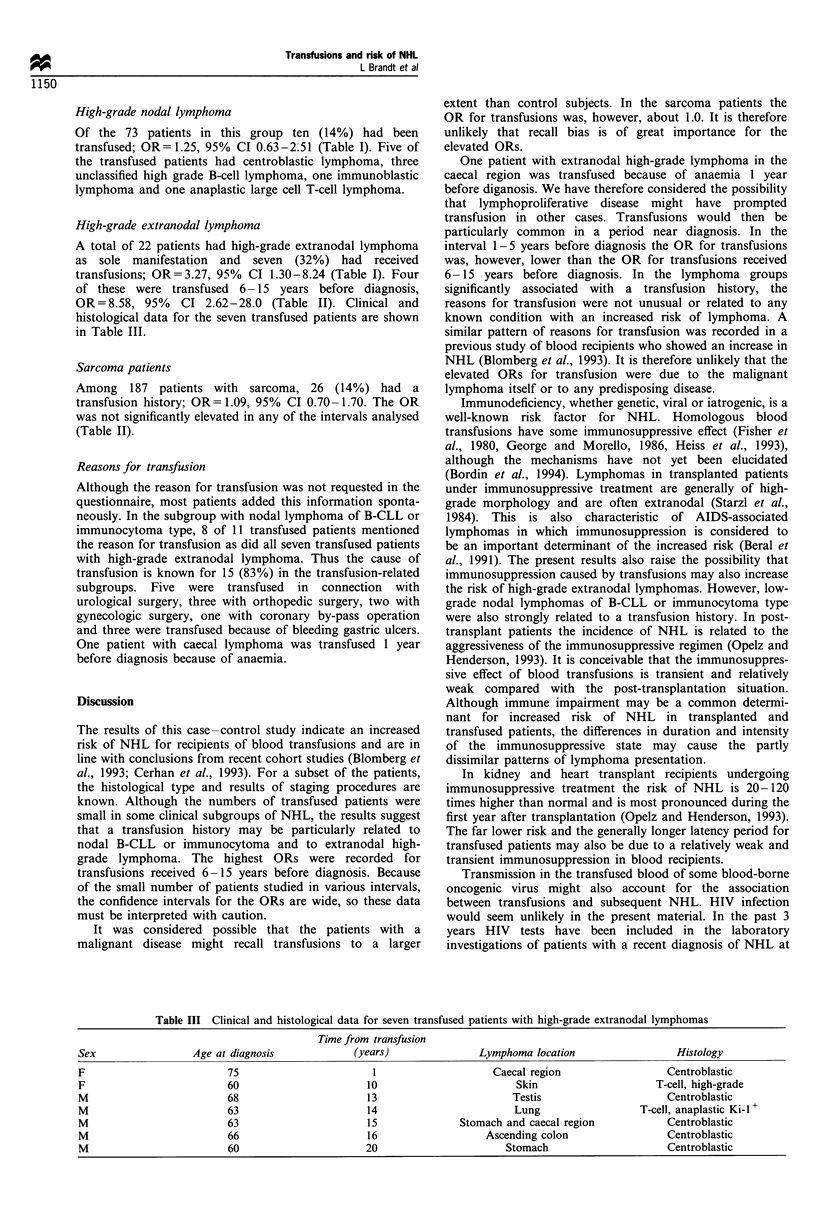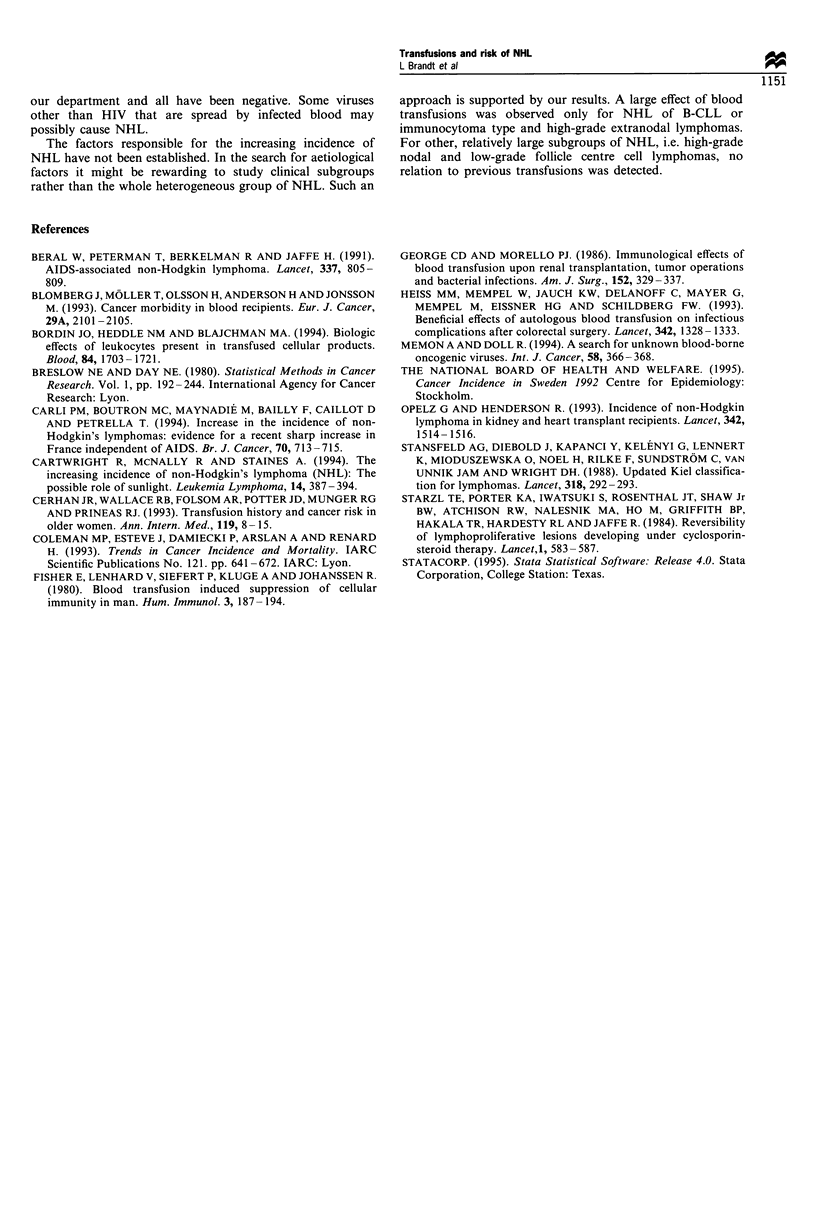# Blood transfusion as a risk factor for non-Hodgkin lymphoma.

**DOI:** 10.1038/bjc.1996.220

**Published:** 1996-05

**Authors:** L. Brandt, J. Brandt, H. Olsson, H. Anderson, T. Möller

**Affiliations:** Department of Oncology, University Hospital, Lund, Sweden.

## Abstract

In a case-control study of 280 out of 426 consecutive patients with a recent diagnosis of non-Hodgkin lymphoma (NHL) and 1827 control subjects, 53 (19%) and 230 (13%) respectively had received blood transfusions 1 year or more before the interview. Using an age- and sex-stratified analysis the odds ratio (OR) for transfusion was 1.74 (95% CI 1.24-2.44). ORs were also determined for transfusions received in the intervals 1-5, 6-15, 16-25 and > or = 26 years before diagnosis. In the interval 6-15 years, the OR for transfusion was 2.83 (95% CI 1.60-4.99) whereas ORs for transfusions received in other intervals were lower and not significantly elevated. Histological diagnoses (Kiel classification) and results of staging procedures were known for 185 patients. For low-grade NHL of nodal B-cell chronic lymphocytic leukaemia (B-CLL) or immunocytoma type, the OR for transfusions was 4.15 (95% CI 1.92-9.01). For low-grade nodal lymphomas of follicle centre cell type and high-grade nodal lymphomas, no relation to transfusions could be demonstrated. For high-grade extranodal lymphoma as sole manifestation, OR for transfusions was 3.27 (95% CI 1.30-8.24). It is concluded that blood transfusion may be a risk factor for NHLs especially those of B-CLL or immunocytoma type and for high-grade extranodal lymphoma.


British Journal of Cancer (1996) 73, 1148–1151
© 1996 Stockton Press All rights reserved 0007–0920/96 $12.00

### Blood transfusion as a risk factor for non-Hodgkin lymphoma

L Brandt[1], J Brandt[2], H Olsson[1], H Anderson[2] and T Möller[2]

[1]Department of Oncology and [2]Southern Swedish Regional Tumour Registry, University Hospital, S-221 85, Lund, Sweden.

**Summary** In a case–control study of 280 out of 426 consecutive patients with a recent diagnosis of non-Hodgkin lymphoma (NHL) and 1827 control subjects, 53 (19%) and 230 (13%) respectively had received blood transfusions 1 year or more before the interview. Using an age- and sex-stratified analysis the odds ratio (OR) for transfusion was 1.74 (95% CI 1.24–2.44). ORs were also determined for transfusions received in the intervals 1–5, 6–15, 16–25 and ⩾26 years before diagnosis. In the interval 6–15 years, the OR for transfusion was 2.83 (95% CI 1.60–4.99) whereas ORs for transfusions received in other intervals were lower and not significantly elevated. Histological diagnoses (Kiel classification) and results of staging procedures were known for 185 patients. For low-grade NHL of nodal B-cell chronic lymphocytic leukaemia (B-CLL) or immunocytoma type, the OR for transfusions was 4.15 (95% CI 1.92–9.01). For low-grade nodal lymphomas of follicle centre cell type and high-grade nodal lymphomas, no relation to transfusions could be demonstrated. For high-grade extranodal lymphoma as sole manifestation, OR for transfusions was 3.27 (95% CI 1.30–8.24). It is concluded that blood transfusion may be a risk factor for NHLs especially those of B-CLL or immunocytoma type and for high-grade extranodal lymphoma.

**Keywords:** transfusion; risk factor; non-Hodgkin lymphoma

The incidence of non-Hodgkin lymphoma (NHL) is increasing quite rapidly in many populations, including Scandinavia (Coleman *et al.*, 1993, Carli *et al.*, 1994, Cartwright *et al.*, 1994, National Board of Health and Welfare, 1995). The causes of this increase are unknown, and in an attempt to identify risk factors for NHL a case–control study has been initiated in the Southern Health Care Region of Sweden.

According to cohort studies of cancer morbidity in blood recipients, transfusions may be a risk factor for NHL. In a Swedish study (Blomberg *et al.*, 1993) an excess of NHL was observed in previously transfused subjects, the standardised morbidity ratio being 4.09 (95% CI 1.65–8.43). Cerhan *et al.* (1993) obtained information about past transfusions from women aged 55–69 years who were resident in Iowa and followed up for 5 years. For women who had ever received a blood transfusion the relative risk of NHL was 2.20 (95% CI 1.35–3.58). Memon and Doll (1994) identified nearly 13 000 infants who received blood transfusions shortly after birth in England, Wales and Scotland and recorded the incidence of subsequent malignant tumours. The incidence of NHL at 15–49 years of age was about twice that expected, but the excess was not statistically significant.

Our ongoing case–control study offered an opportunity to further evaluate the relation between previous blood transfusions and the development of NHL. We report here on the association between transfusions and NHL. The relation between the time lapse since transfusion and the risk of lymphoma has also been studied.

### Subjects and methods

### *Patients*

All patients in the Southern Health Care Region of Sweden with a recent diagnosis of malignancy are reported to the Southern Swedish Regional Tumour Registry. Starting in 1991 adult patients with NHL are asked to complete a questionnaire containing items regarding previous diseases, family history of disease, medication, various life-style factors, occupations. The questionnaire contains an item asking 'Have you ever had a blood transfusion?' If the answer is yes, the patient is asked to state the year of transfusion. The reason for the blood transfusion is not asked. At the time of compiling this report the questionnaire has been mailed to 426 NHL patients and 298 (70%) have responded. Of these, 184 are men and 114 women aged 17–92 years (median 63). Of the responding patients, 18 (6%) were excluded from the analyses because of incomplete answers concerning transfusions, leaving 280 for analysis. The case records for the responding patients have been searched for and at present histological type of lymphoma and the results of the staging procedures are known for 185 patients. The Kiel classification of lymphomas is used (Stansfeld *et al.*, 1988). In our region a uniform programme for the diagnosis, staging and treatment of NHL is followed. The few relevant pathologists already participate in the programme and a histological review of the lymphomas was not considered necessary. Four categories of NHL were studied:

1 low-grade nodal with or without advanced disease;
2 low-grade extranodal;
3 high-grade nodal with or without advanced disease;
4 high-grade extranodal.

In a parallel case–control study the same questionnaire as for the lymphoma patients was mailed to 240 patients with a recent diagnosis of sarcoma, of whom 187 have responded. Their answers concerning transfusions were used to evaluate possible recall bias among patients with malignant tumours.

### *Controls*

Control subjects were selected from the General Population Registry and matched with the lymphoma and sarcoma patients for sex, age and residence. The same questionnaire as the one used for the lymphoma and sarcoma patients was mailed to all controls. About 3500 questionnaires have been mailed and 705 lymphoma controls and 1226 sarcoma controls have answered, of whom 1009 are men and 922 women, their median age being 66 years. Of the responding controls, 104 (5.4%) were excluded from the analyses because of incomplete answers about transfusion, leaving 1827 controls for analysis.

### *Statistical methods*

Odds ratios (ORs) for having received a blood transfusion at any time and in various intervals before the interview were determined by means of multivariate logistic regression

Correspondence: L Brandt
Received 17 February 1995; revised 10 November 1995; accepted 10 November 1995

(Breslow and Day, 1980). Transfusions received less than 1 year before the interview were not considered because recent transfusions might be due to anaemia caused by the malignant disease. For earlier transfusions the time was categorised into four groups 1–5, 6–15, 16–25 and more than 25 years. Transfusions in these periods were represented by indicator variables and it was then noticed that a few persons were transfused in two or more intervals. All analyses were stratified by sex and 10 year age groups using indicator stratifying variables. Residence was not used because indications for medical procedures, including transfusions, are relatively uniform in the Health Care Region.

Controls in the lymphoma and sarcoma studies with the same values of matching variables are exchangeable, because they have been randomly selected from the same population and have answered the same questionnaire. To improve the statistical power, especially in the lymphoma subgroups, all controls were pooled into one large group in all analyses.

The computer program Stata (StataCorp., 1995) was used for all statistical analyses.

### Results

### *Overall risk of transfusions*

A history of transfusion was admitted or denied by 280 NHL patients and 1827 controls. The numbers of transfused patients and controls were 53 (19%) and 230 (13%) respectively. Using an age- and sex-stratified analysis the OR was 1.74, 95% CI 1.24–2.44 (Table I). For transfusions received 6–15 years before diagnosis the OR was 2.83, 95% CI 1.60–4.99 (Table II). The ratios for earlier or later transfusions were elevated but not statistically significant (Table II).

### *Low-grade nodal lymphoma*

Among the patients for whom histological diagnosis and results of clinical staging were available, 82 had low-grade nodal lymphoma with or without disseminated disease and 18 (22%) of these had been transfused; OR for a transfusion history was 2.02, 95% CI 1.16–3.51 (Table I).

In a small subgroup of 31 patients with lymphoma of B-CLL or lymphoplasmacytoid (immunocytoma) type, 11 (35%) had received transfusions; OR=4.15, 95% CI 1.92–9.01 (Table I). In the interval 6–15 years before diagnosis the OR was 8.12, 95% CI 2.95–22.3, whereas ORs for earlier or later transfusions were slightly but not significantly elevated (Table II).

A total of 44 patients had low-grade lymphoma of follicle centre cell type (centrocytic, centroblastic/centrocytic) and five (11%) had a history of transfusion with a non-significant OR=0.91, 95% CI 0.35–2.36 (Table I). Seven other patients had unclassified low-grade lymphomas, of whom two had received transfusions.

### *Low-grade extranodal lymphoma*

Only eight patients presented with low-grade extranodal lymphoma as the sole initial manifestation and one of these (13%) had received a transfusion (Table I).

**Table I** Number of transfused patients and controls. Odds ratios (OR) for all NHL cases and subgroups and for sarcoma cases as an additional comparison group

| *Diagnosis* | n | *Transfused* n *(%)* | *OR* | *95% CI* |
|---|---|---|---|---|
| All NHL | 280 | 53 (19%) | 1.74 | 1.24–2.44 |
| Low-grade nodal | 82 | 18 (22%) | 2.02 | 1.16–3.51 |
| B-CLL or immunocytoma | 31 | 11 (35%) | 4.15 | 1.92–9.01 |
| Follicle centre cell | 44 | 5 (11%) | 0.91 | 0.35–2.36 |
| Low-grade extranodal | 8 | 1 (13%) | 1.07 | 0.13–9.09 |
| High-grade nodal | 73 | 10 (14%) | 1.25 | 0.63–2.51 |
| High-grade extranodal | 22 | 7 (32%) | 3.27 | 1.30–8.24 |
| Sarcoma | 187 | 26 (14%) | 1.09 | 0.70–1.70 |
| Controls | 1827 | 230 (13%) | | |

**Table II** Odds ratios (95% CI) for transfusions received in various intervals before diagnosis. Number of transfused subjects in parentheses

| *Diagnosis* | *Time before diagnosis (years)* *1–5* | *6–15* | *16–25* | *≥26* |
|---|---|---|---|---|
| All NHL ($n$=280) | 1.67 (0.87–3.21) (13) | 2.83 (1.60–4.99) (19) | 1.55 (0.80–2.99) (12) | 1.45 (0.80–2.60) (15) |
| Low-grade nodal ($n$=82) | 1.50 (0.50–4.50) (4) | 4.90 (2.25–10.7) (9) | 1.22 (0.36–4.13) (3) | 1.55 (0.54–4.47) (4) |
| B-CLL or immunocytoma ($n$=31) | 2.89 (0.87–9.66) (4) | 8.12 (2.95–22.3) (6) | 2.04 (0.44–9.53) (2) | 1.34 (0.17–10.6) (1) |
| Follicle centre cell ($n$=44) | 0 (no transfused) (0) | 2.93 (0.85–10.1) (3) | 0.72 (0.09–5.5) (1) | 0.56 (0.07–4.18) (1) |
| High-grade nodal ($n$=73) | 1.61 (0.48–5.45) (3) | 1.11 (0.26–4.79) (2) | 2.32 (0.80–6.74) (4) | 0.37 (0.05–2.71) (1) |
| Low-grade extranodal ($n$=8)[a] | – | – | – | – |
| High-grade extranodal ($n$=22) | 1.45 (0.17–12.32) (1) | 8.58 (2.62–28.0) (4) | 2.83 (0.60–13.3) (2) | 2.33 (0.51–10.6) (2) |
| Sarcoma ($n$=187) | 1.53 (0.67–3.50) (7) | 1.48 (0.65–3.38) (7) | 0.53 (0.16–1.75) (3) | 1.44 (0.76–2.72) (12) |

[a]Too few cases for analysis.

*High-grade nodal lymphoma*

Of the 73 patients in this group ten (14%) had been transfused; OR = 1.25, 95% CI 0.63–2.51 (Table I). Five of the transfused patients had centroblastic lymphoma, three unclassified high grade B-cell lymphoma, one immunoblastic lymphoma and one anaplastic large cell T-cell lymphoma.

*High-grade extranodal lymphoma*

A total of 22 patients had high-grade extranodal lymphoma as sole manifestation and seven (32%) had received transfusions; OR = 3.27, 95% CI 1.30–8.24 (Table I). Four of these were transfused 6–15 years before diagnosis, OR = 8.58, 95% CI 2.62–28.0 (Table II). Clinical and histological data for the seven transfused patients are shown in Table III.

*Sarcoma patients*

Among 187 patients with sarcoma, 26 (14%) had a transfusion history; OR = 1.09, 95% CI 0.70–1.70. The OR was not significantly elevated in any of the intervals analysed (Table II).

*Reasons for transfusion*

Although the reason for transfusion was not requested in the questionnaire, most patients added this information spontaneously. In the subgroup with nodal lymphoma of B-CLL or immunocytoma type, 8 of 11 transfused patients mentioned the reason for transfusion as did all seven transfused patients with high-grade extranodal lymphoma. Thus the cause of transfusion is known for 15 (83%) in the transfusion-related subgroups. Five were transfused in connection with urological surgery, three with orthopedic surgery, two with gynecologic surgery, one with coronary by-pass operation and three were transfused because of bleeding gastric ulcers. One patient with caecal lymphoma was transfused 1 year before diagnosis because of anaemia.

### Discussion

The results of this case–control study indicate an increased risk of NHL for recipients of blood transfusions and are in line with conclusions from recent cohort studies (Blomberg *et al.*, 1993; Cerhan *et al.*, 1993). For a subset of the patients, the histological type and results of staging procedures are known. Although the numbers of transfused patients were small in some clinical subgroups of NHL, the results suggest that a transfusion history may be particularly related to nodal B-CLL or immunocytoma and to extranodal high-grade lymphoma. The highest ORs were recorded for transfusions received 6–15 years before diagnosis. Because of the small number of patients studied in various intervals, the confidence intervals for the ORs are wide, so these data must be interpreted with caution.

It was considered possible that the patients with a malignant disease might recall transfusions to a larger extent than control subjects. In the sarcoma patients the OR for transfusions was, however, about 1.0. It is therefore unlikely that recall bias is of great importance for the elevated ORs.

One patient with extranodal high-grade lymphoma in the caecal region was transfused because of anaemia 1 year before diganosis. We have therefore considered the possibility that lymphoproliferative disease might have prompted transfusion in other cases. Transfusions would then be particularly common in a period near diagnosis. In the interval 1–5 years before diagnosis the OR for transfusions was, however, lower than the OR for transfusions received 6–15 years before diagnosis. In the lymphoma groups significantly associated with a transfusion history, the reasons for transfusion were not unusual or related to any known condition with an increased risk of lymphoma. A similar pattern of reasons for transfusion was recorded in a previous study of blood recipients who showed an increase in NHL (Blomberg *et al.*, 1993). It is therefore unlikely that the elevated ORs for transfusion were due to the malignant lymphoma itself or to any predisposing disease.

Immunodeficiency, whether genetic, viral or iatrogenic, is a well-known risk factor for NHL. Homologous blood transfusions have some immunosuppressive effect (Fisher *et al.*, 1980, George and Morello, 1986, Heiss *et al.*, 1993), although the mechanisms have not yet been elucidated (Bordin *et al.*, 1994). Lymphomas in transplanted patients under immunosuppressive treatment are generally of high-grade morphology and are often extranodal (Starzl *et al.*, 1984). This is also characteristic of AIDS-associated lymphomas in which immunosuppression is considered to be an important determinant of the increased risk (Beral *et al.*, 1991). The present results also raise the possibility that immunosuppression caused by transfusions may also increase the risk of high-grade extranodal lymphomas. However, low-grade nodal lymphomas of B-CLL or immunocytoma type were also strongly related to a transfusion history. In post-transplant patients the incidence of NHL is related to the aggressiveness of the immunosuppressive regimen (Opelz and Henderson, 1993). It is conceivable that the immunosuppressive effect of blood transfusions is transient and relatively weak compared with the post-transplantation situation. Although immune impairment may be a common determinant for increased risk of NHL in transplanted and transfused patients, the differences in duration and intensity of the immunosuppressive state may cause the partly dissimilar patterns of lymphoma presentation.

In kidney and heart transplant recipients undergoing immunosuppressive treatment the risk of NHL is 20–120 times higher than normal and is most pronounced during the first year after transplantation (Opelz and Henderson, 1993). The far lower risk and the generally longer latency period for transfused patients may also be due to a relatively weak and transient immunosuppression in blood recipients.

Transmission in the transfused blood of some blood-borne oncogenic virus might also account for the association between transfusions and subsequent NHL. HIV infection would seem unlikely in the present material. In the past 3 years HIV tests have been included in the laboratory investigations of patients with a recent diagnosis of NHL at

**Table III** Clinical and histological data for seven transfused patients with high-grade extranodal lymphomas

| *Sex* | *Age at diagnosis* | *Time from transfusion (years)* | *Lymphoma location* | *Histology* |
|---|---|---|---|---|
| F | 75 | 1 | Caecal region | Centroblastic |
| F | 60 | 10 | Skin | T-cell, high-grade |
| M | 68 | 13 | Testis | Centroblastic |
| M | 63 | 14 | Lung | T-cell, anaplastic Ki-1$^{+}$ |
| M | 63 | 15 | Stomach and caecal region | Centroblastic |
| M | 66 | 16 | Ascending colon | Centroblastic |
| M | 60 | 20 | Stomach | Centroblastic |

our department and all have been negative. Some viruses other than HIV that are spread by infected blood may possibly cause NHL.

The factors responsible for the increasing incidence of NHL have not been established. In the search for aetiological factors it might be rewarding to study clinical subgroups rather than the whole heterogeneous group of NHL. Such an approach is supported by our results. A large effect of blood transfusions was observed only for NHL of B-CLL or immunocytoma type and high-grade extranodal lymphomas. For other, relatively large subgroups of NHL, i.e. high-grade nodal and low-grade follicle centre cell lymphomas, no relation to previous transfusions was detected.

### References

BERAL W, PETERMAN T, BERKELMAN R AND JAFFE H. (1991). AIDS-associated non-Hodgkin lymphoma. *Lancet*, **337**, 805–809.

BLOMBERG J, MÖLLER T, OLSSON H, ANDERSON H AND JONSSON M. (1993). Cancer morbidity in blood recipients. *Eur. J. Cancer*, **29A**, 2101–2105.

BORDIN JO, HEDDLE NM AND BLAJCHMAN MA. (1994). Biologic effects of leukocytes present in transfused cellular products. *Blood*, **84**, 1703–1721.

BRESLOW NE AND DAY NE. (1980). *Statistical Methods in Cancer Research*. Vol. 1, pp. 192–244. International Agency for Cancer Research: Lyon.

CARLI PM, BOUTRON MC, MAYNADIÉ M, BAILLY F, CAILLOT D AND PETRELLA T. (1994). Increase in the incidence of non-Hodgkin's lymphomas: evidence for a recent sharp increase in France independent of AIDS. *Br. J. Cancer*, **70**, 713–715.

CARTWRIGHT R, MCNALLY R AND STAINES A. (1994). The increasing incidence of non-Hodgkin's lymphoma (NHL): The possible role of sunlight. *Leukemia Lymphoma*, **14**, 387–394.

CERHAN JR, WALLACE RB, FOLSOM AR, POTTER JD, MUNGER RG AND PRINEAS RJ. (1993). Transfusion history and cancer risk in older women. *Ann. Intern. Med.*, **119**, 8–15.

COLEMAN MP, ESTEVE J, DAMIECKI P, ARSLAN A AND RENARD H. (1993). *Trends in Cancer Incidence and Mortality*. IARC Scientific Publications No. 121. pp. 641–672. IARC: Lyon.

FISHER E, LENHARD V, SIEFERT P, KLUGE A AND JOHANSSEN R. (1980). Blood transfusion induced suppression of cellular immunity in man. *Hum. Immunol.* **3**, 187–194.

GEORGE CD AND MORELLO PJ. (1986). Immunological effects of blood transfusion upon renal transplantation, tumor operations and bacterial infections. *Am. J. Surg.*, **152**, 329–337.

HEISS MM, MEMPEL W, JAUCH KW, DELANOFF C, MAYER G, MEMPEL M, EISSNER HG AND SCHILDBERG FW. (1993). Beneficial effects of autologous blood transfusion on infectious complications after colorectal surgery. *Lancet*, **342**, 1328–1333.

MEMON A AND DOLL R. (1994). A search for unknown blood-borne oncogenic viruses. *Int. J. Cancer*, **58**, 366–368.

THE NATIONAL BOARD OF HEALTH AND WELFARE. (1995). *Cancer Incidence in Sweden 1992* Centre for Epidemiology: Stockholm.

OPELZ G AND HENDERSON R. (1993). Incidence of non-Hodgkin lymphoma in kidney and heart transplant recipients. *Lancet*, **342**, 1514–1516.

STANSFELD AG, DIEBOLD J, KAPANCI Y, KELÉNYI G, LENNERT K, MIODUSZEWSKA O, NOEL H, RILKE F, SUNDSTRÖM C, VAN UNNIK JAM AND WRIGHT DH. (1988). Updated Kiel classification for lymphomas. *Lancet*, **318**, 292–293.

STARZL TE, PORTER KA, IWATSUKI S, ROSENTHAL JT, SHAW Jr BW, ATCHISON RW, NALESNIK MA, HO M, GRIFFITH BP, HAKALA TR, HARDESTY RL AND JAFFE R. (1984). Reversibility of lymphoproliferative lesions developing under cyclosporin-steroid therapy. *Lancet*,**1**, 583–587.

STATACORP. (1995). *Stata Statistical Software: Release 4.0*. Stata Corporation, College Station: Texas.